# Calibration of water quality model for distribution networks using genetic algorithm, particle swarm optimization, and hybrid methods

**DOI:** 10.1016/j.mex.2019.03.008

**Published:** 2019-03-16

**Authors:** Roya Peirovi Minaee, Mojtaba Afsharnia, Alireza Moghaddam, Ali Asghar Ebrahimi, Mohsen Askarishahi, Mehdi Mokhtari

**Affiliations:** a Environmental Science and Technology Research Center, Department of Environmental Health Engineering, Shahid Sadoughi University of Medical Sciences, Yazd, Iran; b Department of Environmental Health Engineering, School of Public Health, Gonabad University of Medical Sciences, Gonabad, Iran; c Department of Civil Engineering, University of Gonabad, Gonabad, Iran; d Department of Biostatistics, Shahid Sadoughi University of Medical Sciences, Yazd, Iran

**Keywords:** Genetic Algorithm (GA), Hybrid of Genetic Algorithm and Particle Swarm Optimization (HGAPSO), Particle Swarm Optimization (PSO), EPANET, GA, HGAPSO, PSO, Wall decay coefficient, Water distribution network

## Abstract

Chlorine reacts with both organic and inorganic matters in water. That is why water quality modeling has received great attention in recent years. The serious issue in municipal water quality modeling is gathering the essential input parameters of the model, particularly bulk decay (k_b_) and wall decay (k_w_) coefficients as well as their calibrations. Therefore, this study first thoroughly formulates the problem in the form of a heuristic optimization and then utilizes Genetic Algorithm, Particle Swarm Optimization, and Hybrid GA-PSO as the model optimizers in order to best calibrate k_w_ for minimizing the difference of residual chlorine concentrations that exist between the simulated and observed values. These three algorithms are linked to EPANET, the hydraulic and water quality simulator. The method is then applied to a real-world water distribution network. Here, $k_{w}$ is considered as a decision variable. The objective function is to minimize both the Sum of Square Error and Root Mean Square Error between the observed and simulated chlorine concentrations. According to the simulation results obtained, the optimal value of wall decay coefficient is 1.233 m/day during the calibration process while the minimum and maximum differences between the measured and simulated chlorine rates were 0 and 0.18, respectively.

• The method presented in this article can be useful for managers of water and wastewater companies, water resources facilities and operators and operation manager of water distribution system to manage chlorine dosing rate.
• Due to adverse health effect of disinfection by product and poor microbial water quality as results of inefficient chlorination, control chlorine concentration in water distribution networks and its consequence on human health effect is necessary.
• Hybrid PSO and GA methods are used to cope with their falling in local optimum and requiring highly computational effort.

## Graphical abstract

**Figure:**
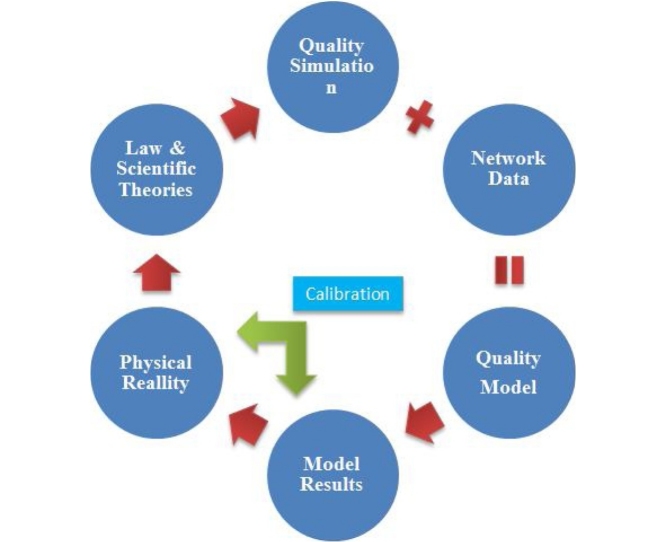

**Specifications Table**

**Table:** 

**Subject Area:**	Environmental modeling Science
**More specific subject area:**	Water quality modeling
**Method name:**	Genetic Algorithm(GA), Hybrid of Genetic Algorithm and Particle Swarm Optimization(HGAPSO), Particle Swarm Optimization(PSO)
**Name and reference of original method:**	- Genetic algorithms in pipeline optimization [1] - A new optimizer using particle swarm theory [2]
**Resource availability:**	- Computer(Intel(R)Core™ i7-7700 3.60-GHz 32 GB - EPANET software [3]

## Method details

### Materials and methods

Hence, in this paper the hybrid method of HGAPSO developed and utilized, which relied on the Genetic Algorithm (GA) and Particle Swarm Optimization (PSO). This method is developed based on a very simple but efficient hybrid use of GA and PSO. Thus, the aim was to calibrate $k_{w}$ to minimize the difference between simulated and observed values of residual chlorine concentrations using EPANET 2.0 as the hydraulic simulator and GA, PSO, and HGAPSO as the optimization tools. The methods were also applied to a real-world water network. Indeed this study investigated the proposed method and compared it with the method used by the previous researchers to perform quality calibration of the network under study. In the following, the methodology employed in this paper, including water quality model, objective function, and optimization algorithms are explained in detail.

#### Water quality modeling

In order to trace the growth or decay of reactions in water pipelines, EPANET requires the rate of the reaction as well as its probably dependence on the substance concentration [4]. Chlorine decay is categorized in two cases of bulk decay: reactions occur in the bulk flow and their rates depend on the concentrations of both organic and inorganic substances and wall decay: reactions occur at or near the pipe wall [3,5,6]. Some studies provided more detailed description of the chlorine decay model [7,8].

Laboratories and field studies have shown that decay constant may vary with respect to a few factors (as previously mentioned). Estimation of the decay constant is a key issue in using the decay equation. Therefore, the total decay constant (K) is often defined as the decay constant composed of two terms, $k_{b}$ and $k_{w}$ [9]:

(1) $$K=k_{b}+k_{w}$$

#### Genetic Algorithm (GA)

Genetic Algorithm is an adaptive search algorithm that works based on the evolutionary ideas of natural selection and genetics. It includes three major operators of selection, crossover, and mutation. This algorithm generates a random initial population (represented by a string of genes or chromosomes) within the search scope. The fitness values of these candidate solutions are assigned proportionally to their pertinent objective function values. Based on fitness values, GA forms a mating pool using the selection operation [10]. The selection process removes the inferior solutions and allows multiple copies of the elite solutions in the mating pool. This step does not create any new solutions. Then, GA performs the crossover operation to generate new solutions. At this stage, the crossover operator randomly picks up two individuals from the mating pool and crosses them to generate two new offspring. However, in order to maintain some of the superior solutions that exist in the parent population, crossover operation will be carried out only in the case that the crossover probability is satisfied. Mutation operation is responsible for maintaining the diversity of the solutions by local altering the genes. Genetic algorithm performs the selection, crossover, and mutation operation in an iterative way until the stop criterion is reached [10].

#### Particle Swarm Optimization (PSO)

Particle swarm optimization (PSO) is a meta-heuristic optimization method that was first used by *Eberhart* and *Kennedy* [2]. This algorithm, inspired by the flocking and schooling of birds and fish belongs to the category of swarm intelligence. In PSO, all possible solutions of a problem are in a search framework called solution space. Each solution in this swarm is called a particle. Each particle, which iteratively flies over the search domain, represents a solution to the problem. Three vectors exist in each iteration that defines the movement of each particle into the next iteration. One of these vectors is the velocity vector, which is randomly generated. The other two vectors update based on the best position of particles. Then, each particle keeps tracking of its position vector; pbest, which has acquired the best fitness function. The position vector; gbestis the best value of fitness function [11]. The position vector of the particles is updated as stated by the velocity vector. These new positions in PSO algorithm are evaluated by an objective or fitness function in each iteration and then pbestand gbestare updated again. The particle velocities $V_{ij}^{t}$and positions $X_{ij}^{t}$ are calculated using the following equations:

(2) $$V_{ij}^{t+1}=wV_{ij}^{t}+c_{1}r_{1}^{t}\left(\text{p}\text{b}\text{e}\text{s}\text{t}\;(ij)-X_{ij}^{t}\right)+c_{2}r_{2}^{t}\left(\text{g}\text{b}\text{e}\text{s}\text{t}\left(\text{j}\right)-X_{ij}^{t}\right)\;$$

(3) $$X_{ij}^{t+1}=X_{ij}^{t}+V_{ij}^{t}$$

Where, i = [1, 2, …, P], j = [1, 2, …, n], $c_{1}$ and $c_{2}$ are acceleration constants that range within [[2], [3], [4]], $r_{1}$ and $r_{2}$ are random numbers that are in the range of 0–1. The gbestand pbest are the global and particle best known positions, respectively. Moreover, w is the initial weight that represents the exploration and exploitation ability of the algorithm and ranges from 0.4 to 0.9. The characteristics of the exploration algorithm increase if w is close to 0.9; otherwise, its exploitation properties increase. In this step, a new modified parameter, called $\;w_{dmp}$ is used in each iteration to enhance the exploration capability during the final steps (Eq. 4). The $w_{dmp}$ factor in this paper is 0.998, which decreases w and particle movement in each iteration [12].

(4) $$w=w\times w_{dmp}$$

#### Hybrid GA and PSO

The advantages of PSO algorithm over GA include its simplicity, intelligibility, and controllability of the convergence rate. In GA, mutation rate and crossover probability influence the algorithm convergence, but it cannot control the rate of convergence as easily as the inertia factor in PSO. The effect of increase in the rate of convergence can be directly observed in PSO as a result of decrease in the inertia factor, but the major restrictions of PSO are its premature convergence and getting stuck in locally optimal points [13]. To avoid this problem, the best position of the swarm should be changed iteratively. In order to hit this target, diversity among the population members can be increased by including the mutation and crossover operators of GA in PSO, so that the probability of falling into local optima is reduced. In HGAPSO, the total number of iterations is specified initially and later the algorithm is divided into its two sub-algorithms of GA and PSO. In the first step, GA generates a high-quality population, in which the solutions are sorted in an ascending order depending on their fitness, where PSO is the best algorithm used for the specific and global purposes in all societies. At the end of each iteration, PSO measures are the best values based on the information provided by GA. These stages continue until the termination conditions are met, or the maximum number of iterations is reached.

#### Optimization model formulation

##### Decision variable

In this paper k_w_ was considered as a decision variable that ranged within 1–1.5 m/day depending on the pipe diameter, material, and initial chlorine concentration [8]. In the proposed model, the wall decay coefficient is adjusted while calibrating the water quality model to reach the least difference between the values obtained by field measurement and simulation. In this regard, the wall decay coefficient can be assigned to study the pipes in three methods: 1) Assigning the same coefficient to all the pipes of the system, 2) Assigning zonal coefficients to the pipes, and 3) Assigning a coefficient a to each pipe that is inversely proportional to the Hazen-Williams roughness coefficient, as stated in Eq. (5), where the fitting coefficient was adjusted during the calibration.

(5) $$K_{w}=\frac{\alpha }{C}$$

In this equation, k_w_ is the wall decay coefficient, α is the fitting coefficient, and c is the Hazen-Williams C-factor [14]. In this study the first method was used in assigning the wall decay coefficient.

##### Objective function

The objective function of the optimization model minimizes the sum of squared error (SSE) and Root Mean Square Error (RMSE) that exist between the observed and simulated chlorine concentrations as described below:

(6) $$Minimize\;\;SSE=\sum_{j=1}^{n}{\left(C_{j}^{obs}-C_{j}^{sim}\right)}^{2}$$

(7) $$Minimize\;\;RMSE=\sqrt{\frac{\sum_{j=1}^{n}{\left(C_{j}^{obs}-C_{j}^{sim}\right)}^{2}}{n}}$$

Where, n is the number of observations $\;\text{a}\text{n}\text{d}\;C_{j}^{obs}$ and $C_{j}^{sim}$ are the observed and simulated chlorine concentrations at junction j mg/L.

**Fig. 1:**
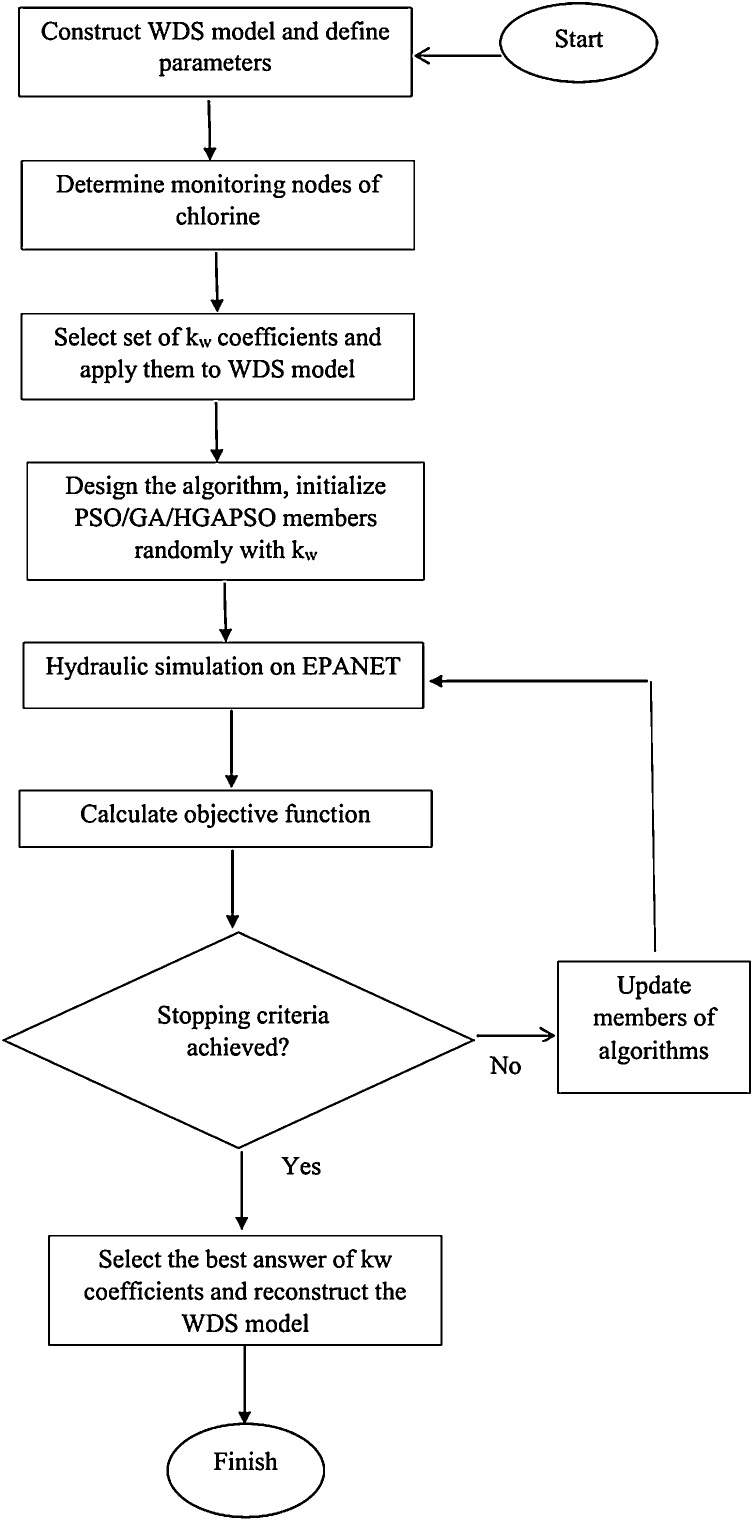
The flowchart of the proposed approach.

The flowchart in Fig. 1 outlines the process of the proposed approach distinctly. The optimization program is first coded in MATLAB and then linked to EPANET 2.0, as the hydraulic simulator.

### Case study

**Fig. 2:**
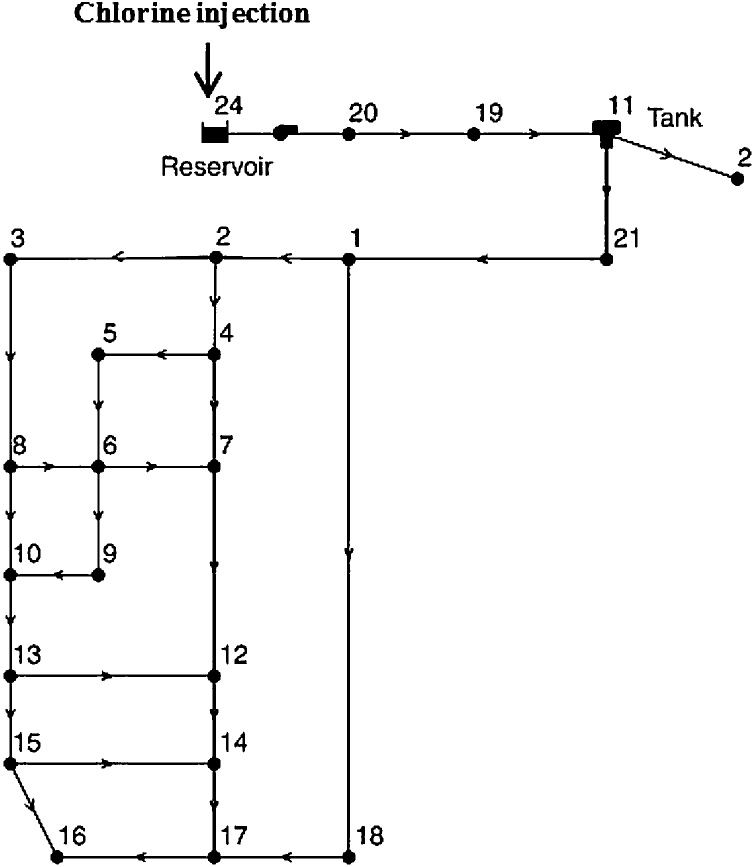
The schema of Miraj DWDS.

In this study, *Miraj* real-world water network was used for calibration and water quality modeling. This water network was previously studied by [8], who applied the inverse model to determine the wall decay. They determined the chlorine reaction rate parameters, so that the observed and computed chlorine concentrations were minimized in a least-squares manner. The region under study included a number of residential houses and apartments. The storage reservoir had a capacity of 1000 m^3^ and a full supply level of 587 m. The dominant pipe material was Cast Iron (CI) with diameters ranging from 80 mm to 400 mm. The schematic representation of this water distribution system is shown in Fig. 2. The chlorine injection dose to the network was the constant value of 1.70 mg/L and $k_{b}$ was considered as 1.73 1/day. Simulation was conducted under the steady-state condition and the demands were constant in nodes during the study time.

### Results and discussion

To model the chlorine transport, DWDS calibration was performed to estimate the wall decay coefficient of Chlorine properly. This goal was achieved using GA, PSO, and HGAPSO algorithms and the results were compared. All these three algorithms aimed at minimizing the objective function. Since in *Munavalli*’s paper, the field-measured chlorine levels were set at 22 nodes [14], specific consideration was given to the same nodes when performing the chlorine concentrations analysis in the present study. Table 1 represents the chlorine levels obtained from the field measurement and simulation given by *Munavalli* et al. along with the values calculated throughout the proposed method. Furthermore, the minimum and maximum differences between the measured and simulated chlorine rates were 0 and 0.18, respectively. Differences of >0.1 mg/L were obtained at seven nodes and none of them exceeded 0.2 mg/L. As Table 1 shows, SSE and RMSE obtained using the proposed methods (GA, PSO, and HGAPSO) were equal to 0.166 and 0.091, respectively. Based on these results, SSE and RMSE reduced by 67.25 and 82.5 percent, respectively compared with *Munavalli*’s method. This indicates that the proposed methods outperformed the previous ones. It should also be mentioned that the results yield $k_{w}$ = 1.233 m/day during the calibration process.

**Table 1:** The calibration data of wall decay coefficient.

Chlorine Location	Observed Residual Chlorine	Simulated Residual Chlorine	
		Calibration methods	
		*Munavalli* et al.	PSO/GA/HGAPSO
1	1.4	1.170	1.316
2	1.1	1.160	1.226
3	1	1.080	1.156
4	1	1.100	1.032
5	0.95	0.920	0.887
6	0.85	0.850	0.771
7	0.9	1.060	0.882
8	0.8	0.960	0.981
9	0.75	0.670	0.659
10	0.8	0.830	0.778
11	1.5	1.300	1.500
12	0.7	0.850	0.755
13	0.7	0.810	0.663
14	0.6	0.760	0.606
15	0.7	0.760	0.575
16	0.6	0.670	0.534
17	0.7	0.640	0.854
18	1.1	0.720	1.056
21	1.3	1.250	1.422
22	1.3	0.960	1.334
Mean ± SD	0.93 ± 0.26	0.92 ± 0.19	0.94 ± 0.28
SSE		0.507	0.166
RMSE		0.520	0.091

**Fig. 3:**
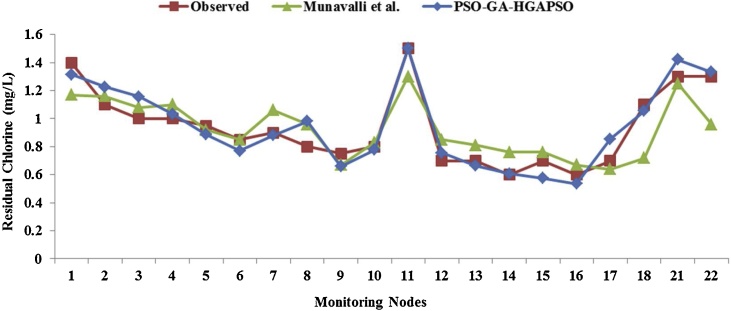
Comparison between the observed and simulated residual chlorine rates in the monitoring nodes.

Fig. 3 represents the two different Chlorine levels at under-monitoring nodes obtained from field observation and simulation by *Munavalli*. It also shows the levels obtained by our simulation-based model. According to this figure, chlorine curve obtained using the proposed models (GA, PSO, and HGAPSO) had fewer mismatches with the field-observed data, especially at nodes: 7, 12, 13, 14, 16, 18, and 22, in comparison with the curve proposed by *Munavalli*.

**Fig. 4:**
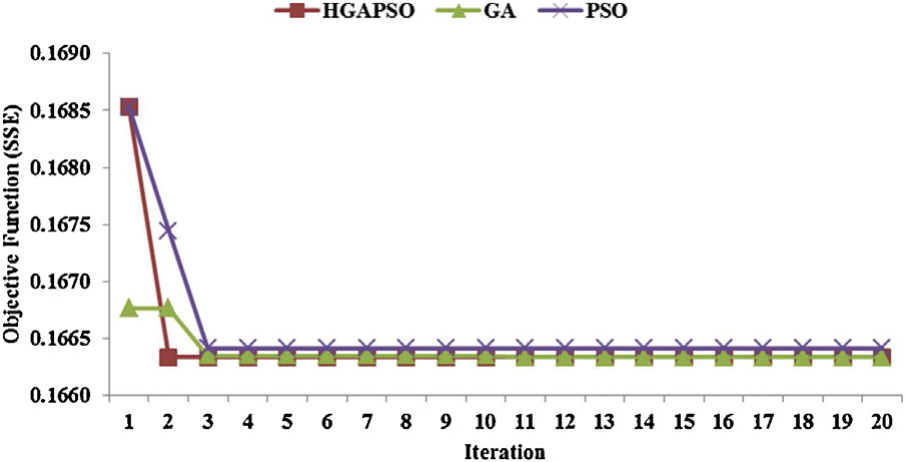
Convergence chart of GA, PSO, and HGAPSO algorithms in Miraj network calibration.

Convergence curves of the three utilized methods are represented in Fig. 4. In order to avoid the random effect of the initial population on the convergence, the initial population was considered the same for all three algorithms. As depicted in Fig. 3, PSO had a fastest convergence. As it can be observed, SSE reduced by 1.33 percent only in two iterations. However, in GA, it reduced by 0.54 percent in five iterations, which represents the global search capability of GA despite its slow convergence rate. Furthermore, we found that HGAPSO reduced SSE by 0.54 percent only in three iterations, indicating that HGAPSO outperformed both GA and PSO. It is worth mentioning that the water network under study (Fig. 2) was not so complicated, which justifies lack of any significant change in SSE after the fifth iteration.

Values of the setting parameters of GA and PSO measured by the sensitivity analysis. The PSO properties include number of population = 10, maximum iteration = 20, *w* = 0.9, *wdamp* = 0.998, as well as $c_{1}$ and $c_{2}$ = 2.05. The GA properties include number of population = 10, maximum iteration = 20, crossover probability, mutation probability and mutation rate are 0.8, 0.3 and 0.01 respectively. Roulette Wheel and Uniform are used as selection and crossover method. In HGAPSO, the parameters were set according to the parameters used in GA and PSO.

### Concluding remarks

Calibration of a water distribution network is beneficial for the operation and control of the water system. In this paper, the water quality modeling and calibration were performed on a real-world drinking water distribution network (Miraj) [8]. In this regard, two objective functions were defined. A novel hybrid optimization algorithm, referred to as HGAPSO was used along with GA and PSO algorithms to optimize these functions. The performance of these methods was evaluated and compared with the findings of previous study. The results clearly demonstrated that the proposed method, i.e., HGAPSO outperformed the other two algorithms. Furthermore, the specific features and advantages of each optimization algorithm were described comparatively. It was observed that the convergence rate of PSO was significant; however, getting stuck was less probable in the local optimum points. In GA, the diversity of generations was wider, although the convergence rate was lower in comparison with PSO. Finally, it was concluded that HGAPSO, as a hybrid of GA and PSO, removed the restrictions of the constituent algorithms, so that the resulting hybrid algorithm was more successful in finding the globally optimal solution. As verified by the results, only a minor difference was observed between the observed and simulated chlorine concentration values. Moreover, wall decay coefficient was obtained 1.233 m/day during the calibration process.

## Conflict of interest

The authors of this article declare that they have no conflict of interests.
